# Chemical Characteristic and Sensory Evaluation of Biscuit Prepared from Wheat and Aleppo Pine Seeds Flour

**DOI:** 10.3390/foods13152428

**Published:** 2024-08-01

**Authors:** Mouni Saadoudi, Adel Lekbir, Omaima Aidat, Sara Zidani, Radhia Ferhat, Dmitry E. Kucher, Timur I. Shiyapov, Nazih Y. Rebouh

**Affiliations:** 1 Food Science Laboratory (LSA), Department of Food Engineering, Institute of Veterinary and Agricultural Sciences, University Batna 1—Hadj Lakhdar, Batna 05000, Algeria; mouni.saadoudi@univ-batna.dz (M.S.);; 2 Laboratory of Food Technology and Nutrition, University of Abdelhamid Ibn Badis, Mostaganem 27000, Algeria; 3 Department of Environmental Management, Institute of Environmental Engineering, RUDN University, 6 Miklukho-Maklaya St., Moscow 117198, Russia; kucher-de@rudn.ru (D.E.K.);

**Keywords:** wheat flour, biscuits, biscuits with Aleppo pine seeds, sensory characteristics, physicochemical features

## Abstract

This study aimed to explore the potential use of Aleppo pine seed flour as a nutritious ingredient in biscuit production. Aleppo pine seed flour was blended with wheat flour to create biscuits with varying proportions: 15%, 30%, and 45% Aleppo pine seed flour. The analysis of the chemical composition of the biscuits revealed that increasing the proportion of Aleppo pine seed flour led to higher levels of protein, fat, and ash, while the carbohydrate content decreased. Sensory evaluation showed that biscuits with 15% Aleppo pine seed flour and 85% wheat flour had favorable characteristics in terms of color, flavor, crispness, and overall appeal. These findings indicate that incorporating Aleppo pine seed flour into wheat flour can enhance the nutritional profile of biscuits, offering higher protein, fat, and mineral content. The study suggests that a blend containing 15% Aleppo pine seed flour is optimal for producing biscuits with desirable sensory properties, making it a promising alternative ingredient for healthier biscuit formulations. Overall, this research highlights the potential of Aleppo pine seed flour to improve the nutritional quality of biscuits without compromising their sensory qualities, paving the way for its use in health-conscious baking practices.

## 1. Introduction

Biscuits hold a prominent position among bakery items on a global scale. This is attributed to their convenience as a ready-to-eat option, affordability, rich nutritional content, diverse range of flavors and tastes, and an extended period during which they remain edible [1]. The characteristics of biscuits depend on the types and quantities of ingredients used. Biscuits are abundant in carbohydrates, fats, and calories, while being relatively deficient in protein, fiber, vitamins, and minerals. A current trajectory within the bakery sector involves creating enhanced biscuits or similar baked goods from composite flours. The surge in attention toward bakery items stems from their improved nutritional profiles and the potential they offer [2].

Biscuits with added protein content are designed to cater to specific groups such as vegetarians and vegans, who often face limited options for obtaining high-quality protein from their diets [3]. In the domain of protein sources, Aleppo pine seeds hold significance as a vital dietary element. They serve as calorie and protein-rich nourishment, encompassing constituents that play crucial roles in biological functions [4].

Aleppo pine *(Pinus halepensis* Mill.), described by Miller in 1768, is a circum-Mediterranean species [5]. Throughout this century, extensive cultivation of *P. halepensis* Mill took place in the Western Mediterranean Basin. However, its focal point appears to be currently shifting to Northern Africa, particularly in Algeria and Tunisia. These regions contain the largest concentration of the plant due to its exceptional ability to withstand drought conditions, with *P. halepensis* Mill. being the dominant species. The tree known as “Snouber” holds the distinction of being the prevalent tree in Algeria, covering an approximate land expanse of 900,000 hectares. It thrives at altitudes reaching up to 2000 m from the coastal regions [6]. *P. halepensis* Mill. is categorized within the diploxylon pine group, recognized for yielding the world’s most extensively traded pine seeds. Traditional medicine utilizes these plants as natural remedies for various ailments, including their capacity to address fatigue and contribute to anti-aging effects [7], possessing properties such as anti-inflammatory, antineoplastic, and antibacterial effects [6,8]. 

As a matter of fact, the seeds ensconced within substantial cones boast a wealth of nutrients, making them a fundamental element integrated into a wide array of traditional culinary preparations [5]. Additionally, Tukan et al. [4] demonstrated that Aleppo pine seeds possess elevated levels of carbohydrates and protein. Among the principal amino acids, glutamic acid (5.5% of dry weight) and arginine (4.0%) dominate, together constituting about one third (33%) of the seed’s protein content. Storage proteins typically exhibit a notable abundance of arginine, which is the amino acid with the highest nitrogen content.

The oil extracted from Aleppo pine seeds exhibits a substantial oil content (approximately 40%), indicating its considerable potential as a vegetable fat source [9]. According to findings by Nasri et al. [10], the primary fatty acid present is linoleic acid, constituting the majority (56.06%) of the total fatty acid composition, followed by oleic acid (24.03%) and palmitic acid (5.23%). The oil extracted from these seeds is recognized as a valuable source of natural antioxidants. Moreover, Cheikh-Rouhou et al. [11] underscored the presence of significant mineral elements within Aleppo pine seeds. 

Over the centuries, numerous Arabic countries employed *P. halepensis* seeds in the preparation of a sweet pudding referred to as “Assida-Zgougou”. More recently, they were incorporated into ice creams and candies [5]. In Jordan and Palestine, the seeds are consumed as snacks, either in their raw state or after being roasted. They feature prominently in various traditional dishes. Particularly, they form a key component of “Malban fruit leather”, a confection made from semolina thickened with concentrated grape juice, notably produced in the Palestinian town of Hebron. Additionally, these seeds find their way into whole wheat porridge and are liberally utilized in the creation of the traditional and popular yeast-leavened bread known as “Quzmat”. This bread is crafted from whole wheat flour, olive oil, and an assortment of oil seeds [4]. In Algeria, Aleppo pine seeds are primarily used to prepare “Tamina”, a mixture of wheat and Aleppo pine seeds, and are also employed to decorate a type of biscuit known as “Ghribia”.

Nevertheless, limited research explored the potential of enhancing wheat flour with Aleppo pine seed flour for biscuit production. Recognizing the nutritional value and health advantages of Aleppo pine seeds, this study aims to introduce Aleppo pine seed flour as an innovative and wholesome ingredient to develop high-protein biscuits. The focus is on evaluating the impact of incorporating Aleppo pine seed flour on the physicochemical attributes and sensory qualities of the resulting biscuits.

## 2. Materials and Methods

### 2.1. Sample Preparation

Aleppo pine seeds (about 10 kg) were acquired from an herbal market situated in Merouana, located within the Aures region of Algeria. These seeds were subjected to a cleaning process to remove any foreign matter and then stored at room temperature for a period not exceeding 4 days. A quantity of 1 kg of seeds was ground using a Délonghi coffee grinder, the resulting ground seeds underwent a sieving process using a 1–2 mm sieve, and the obtained product was then stored in airtight bags at a temperature of −20 °C until analysis.

### 2.2. Fundamental Physicochemical Analysis of Aleppo Pine Seeds

The physical properties of Aleppo pine seeds (seed index: weight of 1000 seeds in grams) and bulk density (g/cm^3^) of Aleppo pine seeds were estimated according to the method of Kadri et al. [6]. The proximate composition was determined according to the Association of Official Analytical Chemists’ methods (AOAC) [12]. Samples were dried at 105 °C to a constant weight to determine content of moisture. Crude protein content was determined by the Kjeldahl method using a conversion factor of 6.25. Crude fat (as ether extract, EE) content was determined by the Soxhlet method with petroleum ether used as a solvent. Crude ash content was determined by incineration at 550 °C. The cations and microelements (calcium, magnesium, iron, zinc, copper, phosphorus, and manganese) were determined by atomic absorption spectrophotometry (Agilent AA240 Fs) equipped with air–acetylene, while the concentration of sodium and potassium was determined via a flame photometer. The carbohydrate content was evaluated using the phenol–sulfuric acid colorimetric method, as detailed by Dubois et al. [13]. All analyses were carried out in triplicate. 

### 2.3. Determination of Phenolic Compounds in Aleppo Pine Seeds

#### 2.3.1. Extraction of Phenolic Compounds

A quantity of one gram of Aleppo pine seeds powder was subjected to extraction using 40 mL of methanol at ambient temperature during 24 h with continuous agitation. After undergoing centrifugation and filtration, the resulting extracts were concentrated under reduced pressure at 40 °C using a rotary evaporator (Büchi, Switzerland). Subsequently, the concentrated extracts were reconstituted in 10 mL of the same solvent.

#### 2.3.2. Determination of Total Phenolic

The assessment of total phenolic compounds was conducted through the Folin–Ciocalteu method, following the protocol outlined by Singleton et al. [14], with certain adaptations. A diluted extract (0.5 mL) was combined and mixed with 2.5 mL of Folin–Ciocalteu reagent. After 3 min, the mixture was supplemented with 2 mL of Na_2_CO_3_ (7.5%). The final mixture was shaken and then incubated for 1 h in the dark at room temperature. The absorbance of all samples was measured at 765 nm utilizing a Spectrophotometer (Beckman 34 UV-Vis, Brea, CA, USA). The calibration curve was prepared with a gallic acid standard in different concentrations.

#### 2.3.3. Estimation of Total Flavonoids Content

Total flavonoid content in the extracts was estimated using the spectrophotometric procedure detailed by Maksimovic et al. [15]. This method relied on the creation of a flavonoid–aluminum complex. Specifically, 1 mL of a diluted sample was mixed with 1 mL of a 2% methanolic solution containing aluminum chloride. Following an incubation period at ambient temperature lasting 10 min, the absorbance of the resulting mixture was measured at 415 nm. To establish the calibration curve, quercetin was used as a reference standard.

### 2.4. Preparation of the Aleppo Pine Seed Flour

The Aleppo pine seeds were subjected to a thorough rinsing with distilled water, then they were dried in the oven (Memmert SLE 400, Schwabach, Germany) at 60 °C for a duration of 12 h. Subsequently, the dried seeds were milled into a powder and passed through a 300 μm sieve to achieve a finely textured consistency. The resulting flour was then carefully stored in plastic containers at room temperature.

### 2.5. Biscuit Preparation

Four distinct formulations of Aleppo pine seeds biscuits were prepared, each of them differs in the ratio of wheat to Aleppo pine seed flour as outlined in Table 1. The basic formulation consisted of 100 g of flour, 45 g of fat, 40 g of sugar, 30 g of egg, and 1 g of baking powder. The process involved adding the fat to the dough mixer and stirring for a brief period. Subsequently, the egg and sugar were introduced and mixed for a duration of 2 min. Following this, the flour and baking powder were added, and the mixture was mixed for an additional 5 min. The resulting batter was shaped using a biscuit cutter and then baked in an oven at a temperature of 180 °C for a period of 20 min.

### 2.6. Identification of Fundamental Constituents of the Biscuits

The content of moisture, crude protein, crude ash, and crude fat was determined according to the Association of Official Analytical Chemists (AOAC) [12]. Meanwhile, the carbohydrate content was assessed using the phenol–sulfuric acid colorimetric technique outlined by Dubois et al. [13]. The calculation of energy value involved summing the products of crude protein, fat, and carbohydrate values with their corresponding Atwater factors (4, 9, and 4, respectively). The assessment of total phenolic compounds was conducted through the Folin–Ciocalteu method, following the protocol outlined by Singleton et al. [14]. Total flavonoid content in the extracts were estimated using the spectrophotometric procedure detailed by Maksimovic et al. [15].

### 2.7. Determination of Physical Properties of the Biscuits

The dimensions of the biscuits, including both their thickness (height) and diameter, were measured using a digital vernier caliper, following the approach outlined by Korus et al. [16]. The spread ratio of the biscuits was determined by dividing the diameter of the biscuit by its thickness, employing the formula as indicated by Wang et al. [17]. Color was measured using a color reader, Minolta CR 10 (Minolta Camera, Osaka, Japan). Results are expressed according to CIELAB system (L*, a*, and b*).

### 2.8. Sensory Evaluation

A trained panel composed of 25 members carried out a quantitative descriptive sensory analysis on the biscuits, following the protocols described in a prior study outlined by Pasqualone et al., [18]. The evaluation encompassed attributes such as color, taste, crispness, appearance, and overall acceptability, employing a 9-point hedonic scale. In this scale, a rating of 9 corresponded to “like extremely”, 8 to “like very much”, 7 to “like moderately”, 6 to “like slightly”, 5 indicated “neither like nor dislike”, 4 represented “dislike slightly”, 3 signified “dislike moderately”, 2 was “dislike very much”, and 1 indicated “dislike extremely”.

### 2.9. Statistical Analysis

Statistical analysis was carried out using SPSS statistical software (Version 21.0). To assess the significance of differences between mean values at a significance level of *p* < 0.05, the Duncan’s test was employed. Furthermore, principal component analysis (PCA) was conducted on selected physicochemical parameters of the biscuits.

## 3. Results and Discussion

### 3.1. Chemical Properties of Aleppo Pine Seeds

Table 2 provides an overview of some physical and chemical attributes of Aleppo pine seeds.

The average values of the index of the seeds and the bulk density of *P. halepensis* seeds, cultivated in Algeria are represented in Table 2. The index of the seeds and the bulk density in Aleppo pine seeds were 15.43 g and 0.39 (g/cm^3^), respectively. These results are lower than those (51.00 g and 0.52 g/cm^3^) reported, respectively, by Kadri et al. [6] for Aleppo pine seeds from the same country. However, the obtained result for the index of the seeds is comparable to the value (17.7 g) reported by Schiller and Waisel [19] for Aleppo pine seeds cultivated in Israel. The difference in the values found of the index of the seeds and the bulk density of Aleppo pine seeds could be related to the difference in the ecological environment and the genotype and phenotypic variability [6]. The seed index and the bulk density of *P. halepensis* seeds is a useful tool for the assessment of the potential milling yield. On the other hand, Khan et al. [20] indicated that the seeds possessing a higher seed index and higher bulk density present a better potential for grinding and flour extraction.

As indicated in Table 2, the moisture content registers at 5.6 g/100 g. A parallel outcome was observed by Cheikh-Rouhou et al. [11] for *P. halepensis* cultivated in Tunisia (5.2 g/100 g). However, there is a slight difference when compared to the findings of Kadri et al. [6] for *P. halepensis* cultivated in Algeria (7.86 g/100 g). This variance could potentially be attributed to differences in seed maturation, cone age, climatic conditions of the harvest site, drying technique, and storage conditions of the seeds. The overall moisture content of the examined *P. halepensis* seeds falls within a comparable range to that of other intact dry seeds, such as *P. pinea*, *P. pinaster*, and *P. canariensis*, which all exhibit humidity levels around 7–9 g/100 g [6]. 

The Aleppo pine seeds cultivated in the Merouana region of Algeria exhibit a protein content of 28.34 g/100 g. This outcome closely aligns with the protein content discovered in Jordanian *P. halepensis* seeds (29.8 g/100 g) as reported by Tukan et al. [4]. However, Cheikh-Rouhou et al. [11] found a protein content of 22.7 g/100 g in Tunisian *P. halepensis* seeds. In comparison to leguminous seeds such as dry beans, lentils, and chickpeas, or cereal grains such as wheat, Aleppo pine seeds could be categorized as protein-rich seeds. They can be ranked alongside cereals and legumes due to their noteworthy protein content, serving as significant sources of nutrition abundant in essential amino acids and nitrogen [6]. Vegetable proteins are a very good alternative to animal proteins, because of their low cost, abundance and diversity of their sources (legumes, cereals, and oilseeds), their adequate quality and nutritional value, their ease of digestion, their non-toxicity, and finally for their functionality [21].

As shown in Table 2, the carbohydrate content of *P. halepensis* is recorded at 24.81 g/100 g. This outcome closely aligns with the carbohydrate content discovered for Tunisian *P. halepensis* (25.7 g/100 g) by Cheikh-Rouhou et al. [11]. The substantial presence of soluble sugars in these seeds contributes to a notable caloric intake. Additionally, Kadri et al. [6] noted that the main sugar found in these seeds is sucrose.

The seeds constitute a substantial source of lipid, which represents 37.26 g/100 g of their composition. Compared to seeds grown in other regions, the oil content is greater than that reported by Bagci et al. [22] (21.1 g/100 g) in seeds of Turkish pines and slightly higher than that reported by Tukan et al. [4] (32.1 g/100 g), Al-Ismail et al. [23] (31.25 g/100 g) in seeds of Aleppo pine cultivated in Jordan and Khouja et al., [24] (33.7 g/100 g) in Tunisian Aleppo pine seeds, and less than that reported by Cheikh-Rouhou et al. [11] (43.3 g/100 g) for *P. halepensis* Mill. cultivated in Tunisia. These seeds offer a promising reservoir of essential fatty acids, making them a valuable energy source. In a broader context, pine seeds generally exhibit rich lipid content, with variations due to species differences, geographical influences, and environmental factors [25,26]. Compared to other oleaginous seeds such as sunflower and sesame, *P. halepensis* seeds have a relatively lower lipid content [6]. On the contrary, the lipid content in soybean seeds is less than that found in pine seeds [27].

As presented in Table 2, the total phenol content was 663.23 mg/100 g in *P. halepensis* Mill., while the flavonoid content reached 28 mg/100 g. The obtained result of phenolic compounds is greatly exceeding the values reported by Salim et al. [28] (479.6 mg/100 g) in Palestinian Aleppo pine seeds, Kadri et al. [6] (371 mg/100 g) for Algerian *P. halepensis* seeds and Dhibi et al. [9] (72 mg/100 g) in Aleppo pine seeds collected from Tunisia. These results are in agreement with those obtained by Nasri and Triki [29], who revealed that pine species grown in northern Algeria show significantly higher phenolic compound concentrations than those grown in Tunisia. Regarding flavonoids, the obtained content is lower than those found by Salim et al. [28] (82 mg/100 g), Kadri et al. [6] (80 mg/100 g), and Dhibi et al. [9] (35 mg/100 g), respectively, in Palestinian, Algerian, and Tunisian Aleppo pine seeds. A multitude of factors, including growing conditions, geographic origin, fertilizer usage, soil composition, extraction solvents, climate type, provenance, and sunlight exposure, could contribute to these observed differences. In the last decade, there was a lot of focus on the potential health benefits of dietary plant polyphenols as antioxidants. According to epidemiological research, a diet reach in polyphenols protects against cancer, cardiovascular disease, diabetes, osteoporosis, and neurological diseases [30].

Aleppo pine seeds consist of 6.52 g/100 g ash (dry matter) and are considered as a very good source of ash. Results are in line with results reported by Khouja et al. [31]; 7.1 g/100 g while it was higher than that reported by Tukan et al. [4]; 5.6 g/100 g and less than that reported by Cheikh-Rouhou et al. [11]; 8.3 g/100 g. Compared to those of other conifer seeds, the ash content of *Pinus halepensis* seeds was higher than that of *Pinus pinea* (4.5 g/100 g) [25], *Pinus pinaster* (4.62 g/100 g), and *Pinus canariensis* (4.84 g/100 g) [6]. 

The results of mineral contents show also that *P. halepensis* seeds contained significant amounts of important mineral elements (macro and micro constituents). Phosphorus was the most abundant element, followed by potassium, magnesium, and calcium. These elements are vital components of our food and are very important for the maintenance of good health. Other elements (Fe, Na, Zn, Mn, and Cu) were also detected in different amounts (Table 3). The same profile of mineral was found by Kadri et al. [6] but with slightly different values. From a nutrition point of view, *P. halepensis* seeds offer noteworthy concentrations of microelements (Fe, Zn, Mn, and Cu). These elements can help significantly in meeting the daily mineral needs.

### 3.2. Physical Properties of the Developed Biscuits

**Figure 1:**
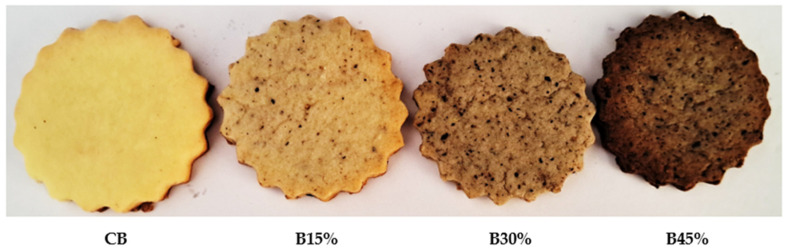
Appearance of biscuits: CB is control biscuit (100% wheat flour), B15%, B30%, and B45% are biscuits, respectively, with 15%, 30%, and 45% Aleppo pine seed flour.

The physical characteristics (thickness, diameter, weight, spread ratio, and color) of the four types of biscuit and their appearance are shown in Table 4 and Figure 1.

As shown in Table 4, changes in the ratio between Aleppo pine seed flour and wheat flour exert a slight perceptible impact on biscuits diameter, thickness, weight, and spread ratio. This is an indication that the biscuits have uniform leavening ability during baking. Compared to the control biscuit, the incorporation of Aleppo pine seed flour did not affect the physical properties of all the composite biscuits (B15%, B30%, and B45%). This observation aligns with the findings of Jisha et al. [32], Devi and Haripriya [33], and Adegbanke et al. [34], which report a slight significant difference in the spread ratio of cookies produced, respectively, from wheat flour and cassava-based composite flours, soy flour, and Bambara groundnut flour. The spread ratio serves as another tangible indicator of biscuit quality, and higher values of this index are appreciated for the positive influence on the acceptability of biscuits [35]. Color is an important quality attribute in the food industry because it has a huge impact on consumer choices and preferences. The supplementation with Aleppo pine seed flour resulted in a relevant increase in red indices, whereas the brown and yellow indices decrease significantly compared to control biscuit. This might due to the color of Aleppo pine seed flour being naturally brown compared to wheat flour.

### 3.3. Chemical Composition of Biscuits

The basic determinations of moisture, carbohydrates, fat, protein, and ash presented in Table 5 give an indication of the nutritive value of the prepared biscuits, but the values of these change depending on the mixture of raw materials used to make the biscuits. 

As seen in Table 5, the moisture content of the biscuit samples ranged from 2.27 to 3.52%, the biscuit B45% displays the lowest value, however the biscuits BC and B30% present the highest values. Baking is a very important process to achieve a good texture and the structure of the biscuits, the oven temperature affects the moisture loss during baking and the evaporation starts from the dough surface, achieving about a 2–4% moisture content in the final products. This process transforms the physical and chemical characteristics of the dough when baked in an oven, where the temperature and time will be accurately controlled. The decrease in moisture level could also mean better shelf stability and potentially limit the growth of microorganisms.

With the exception of carbohydrates, the ash, fat, and protein content of the composite biscuits increased proportionally with increasing incorporation of Aleppo pine seed flour. The results show that the control biscuit had the lowest protein content; however, the protein content of the composite biscuits was significantly influenced by the blending ratio. This was expected because Aleppo pine seed flour contain more protein than cereals, although the prevalent protein in wheat occurs as gluten, which is needed in baking. These results are in good agreement with the observations made by Alobo et al. [36], who showed that the protein content increases with increasing in the rate of substitution of sesame seed flour, which is rich in protein. Wheat flour is low in protein (7 to 14%) and deficient in some amino acids, such as lysine [37]; Aleppo pine seed flour, on the other hand, is higher in protein quantity than wheat flour, but is also deficient almost in all essential amino acids (Tukan et al.) [4]. The combination of wheat flour and Aleppo pine seed flour can be used in the production of biscuits to provide benefits by increasing the dose of protein consumed and supporting certain amino acids which are present in limited quantities. 

The fat content ranged from 29.41% to 35.11%, with the highest value observed in sample B45%. This is attributed to the higher fat content naturally present in Aleppo pine seeds. Algerian *P. halepensis* seeds are notably rich in total oil content, containing around 37.26% (Table 2), which positions them as valuable sources of essential fatty acids and energy. It is also worth noting the role of fats in extending the shelf life of food products.

The ash content of the biscuits demonstrated an increase with the incorporation of Aleppo pine seed flour, potentially due to the high mineral content (6.52%) present in Aleppo pine seed flour (Table 2). From Table 3, it can be seen that *P. halepensis* seed flour contained significant amounts of important mineral elements (macro and micro constituents). These results are consistent with those found by Kadri et al. [6] who demonstrated that *P. halepensis* seeds are rich in appreciable mineral content, notably in phosphorus, potassium, magnesium, calcium, iron, and copper. These elements have important implications for health. The data obtained are broadly consistent with those obtained by Baljeet et al. [38], who showed that the ash content of biscuits exhibited an increase with the addition of high-ash buckwheat flour.

In terms of carbohydrate content, significant differences were observed between the different biscuit formulations. We note that increasing incorporation of Aleppo pine seed flour caused a significant decrease in carbohydrate content, and this decrease can be explained by the low carbohydrate content (24.81%) present in Aleppo pine seed flour (Table 2). These results are in agreement with Aly et al. [39], who demonstrated that the addition of barley flour, which contains a low carbohydrate content compared to wheat flour, could lead to a reduction in the carbohydrate content of composite biscuits. 

The total phenolic compound of biscuits ranged from 3.57 to 31.95 mg/100 g and the flavonoids compound content from 0.24 to 1.50 mg/100 g. The addition of Aleppo pine seed flour to wheat flour resulted in a significant increase in phenolic and flavonoids content of composite biscuits. On the other side, the Aleppo pine seed flour was significantly richer in phenolic and flavonoid compounds than composite biscuits (B15%; B30%; and B45%) and this could be due to the sensitivity of phenolic components to heat. Mahloko et al. [1] indicated that the decrease in phenolic compounds is attributed to the fact that baked products drastically reduce levels of phenolic compounds because of the depolymerization of polyphenols and decarboxylation of phenolic acids that occur during thermal treatment. Moreover, Che Sulaiman et al. [40] indicated that the most phenolic compounds are heat sensitive and easily oxidized, hence an upper limit temperature must be observed to preserve its useful components.

The energy value of the biscuits ranges from 538.03 to 568.11 kcal, the biscuit B15% presents the lowest value; however, the biscuit B45% registers the highest calorie content. This increase in energy value can be interpreted by the richness of the Aleppo pine seed flour in lipids (37.26%) and protein (28.34%). 

The present findings indicate that the incorporation of Aleppo pine seed flour in biscuits has the potential to increase the nutritional value of these products through an augmentation in protein, fat, and ash content, while concomitantly leading to a reduction in carbohydrate content.

**Figure 2:**
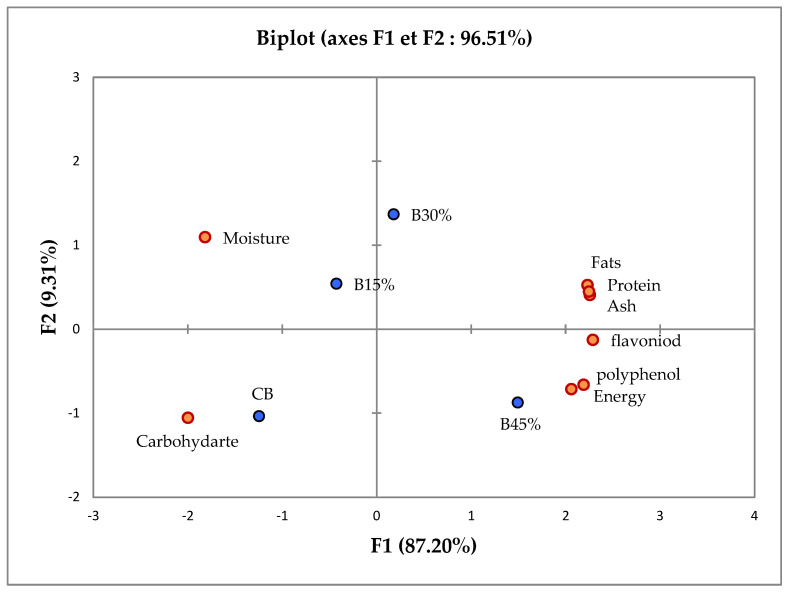
Principal component analysis of physic-chemical characteristic of Aleppo pine seeds enriched biscuits. Where CB is control (100% wheat flour), B15% is blending ratio 1 (15% Aleppo pine seed flour and 85% wheat flour), B30% is blending ratio 2 (30% Aleppo pine seed flour and 70% wheat flour), and B45% is blending ratio 3 (45% Aleppo pine seed flour and 55% wheat flour).

The principal component analysis (PCA) outcomes offer lucid insights into the relationships between physicochemical parameters and the distinctions among the composite biscuit formulations. The PCA captured 87.20% and 9.31% of the variability through PC1 and PC2, respectively (Figure 2). By scrutinizing the correlation matrix, it becomes apparent that a correlation exists between polyphenols, flavonoids, fat, protein, and ash parameters in the physicochemical profile of composite biscuits. Conversely, a discernible separation emerges between the energy value and moisture content of the composite biscuits. According to the first axis, B45% contains the higher energy, fat, protein, ash, polyphenols, and flavonoids levels, while displaying lower carbohydrate and moisture contents. On the contrary, the control biscuit (CB) exhibits elevated carbohydrate content as well as reduced protein, fat, ash, polyphenols, and flavonoids. However, B15% and B30% demonstrate intermediary outcomes. In essence, the current findings underscore that the introduction of Aleppo pine seed flour into biscuits has the potential to enhance the nutritional value of these products through an increase in protein, fat, ash, polyphenols, and flavonoids content, associated with a concurrent reduction in carbohydrate content.

### 3.4. Sensory Properties of Biscuits

The biscuits, prepared from a mixture of Aleppo pine seed flour and wheat flour, were subject to an evaluation based on their color, taste, crispness, appearance, and overall acceptability, utilizing a 9-point hedonic scale (Table 6).

Color represents an essential quality attribute in the field of food industries as it has an immense influence on consumers choice and preferences [41]. The color evaluation scores of composite biscuits decreased from 6.55 to 4.19. The control biscuit (CB) showed the highest score, while the lowest score was given to B 45%. On the one hand, the incorporation of Aleppo pine seed flour changes remarkably the color of the biscuits and makes it darker, which reduces the color preference of consumers, but on the other hand, the increasing of the Aleppo pine seed flour rate significantly improved the protein, lipid, ash, polyphenols, and flavonoids content of the composite biscuits. The color of the biscuits not only indicates the suitability of the raw materials used for preparation, but also provides a cue about the formulation as well as the quality of the end product. These findings are consistent with study by Elkatry et al. [42] and Aksoylu et al. [43], that documented changes in biscuit color brought about by the addition of byproducts such as seeds. On other hand, baking alters also the color of the biscuit surface, this darker appearance in biscuits was attributed to the Maillard reaction between the amino acids and sugars of the biscuits, which is often desired in baked goods [42,44]. These brown pigments are also produced due to the sugar caramelization during the baking process [45]. 

In terms of taste, the subjects felt a more pronounced bitterness in the biscuit containing the highest quantity of flour (B45%), whereas the sweetness was discerned in all other biscuit samples.

Crispness is a force required to break biscuit structure rather than deform it when it is chewed with the human teeth [46]. The crispness scores ranged from 7.12 to 8.31, the B45% showed the highest score, while the lowest score was presented by the control biscuit. The results obtained show a significant increase in the score of crispness as the composite flour supplementation increased, this observation can be attributed to the granular and slightly heterogeneous nature of Aleppo pine seed flour.

The appearance of the product makes the first impression in the consumer’s mind. The average appearance score for our biscuits ranged from 5.27 to 8.51; the highest score was found in the control biscuit (CB), while the lowest score was obtained with B45%. This corresponds to the findings of Khouja et al. [31], who stated that, the most preferred sample is the one which has the smallest quantity of defatted dough of Aleppo pine seeds, unlike the one containing the largest quantity.

The sensory scores for the overall acceptability of the developed biscuits, as well as the control sample, ranged from 5.33 to 7.91. The control biscuit and sample B15% have the best scores than other biscuits samples. The overall acceptability score for the entire biscuit was above five. Meilgaard et al., [47] showed that an overall acceptability score higher than 5 is considered as an acceptable standard score. The evaluation of the sensory quality of the prepared biscuits shows that the control biscuit and sample B15% demonstrated the most preferred choices.

## 4. Conclusions

Biscuits are popular snack foods made from flour, sugar, and fat. Many researchers tried to develop new nutritious products by incorporating new bioactive compounds and different proteins into biscuits. This study highlights the feasibility of producing biscuits with considerable nutritional value. Wheat and Aleppo pine seed flour were combined to produce new nutritious products by incorporating new bioactive compounds and different components such as proteins, fat, and ash into composite biscuits. This substitution has significant potential to solve the problems of protein–energy malnutrition and has a positive biological impact on human health. Aleppo pine seed flour’s addition into biscuit formulation had considerable effects on the physicochemical and sensory properties of biscuits. The sensory analysis showed that the quality of composite biscuits enriched with Aleppo pine seed flour is not comparable to that of wheat biscuits (CB). The overall acceptability sensory outcomes indicate that all the prepared biscuits were accepted by the tasters, and in particular, the B15%. In general, the inclusion of Aleppo pine seeds at low or moderate ratios, results in biscuits with considerable acceptable sensorial. The preparation of biscuits with Aleppo pine seed flour is a good example of valorization of these edible seeds, and the product obtained is highly appreciated and can be widely marketed. However, further studies will be needed to evaluate the dietary fiber content and the biological activities (antioxidant, antibacterial, etc.) of Aleppo pine seeds.

## Figures and Tables

**Table 1:** Ingredients used in Aleppo pine seeds biscuit preparation.

Ingredients (g)	CB	B15%	B30%	B45%
Wheat flour	100	85	70	55
APSF	0	15	30	45
Fat	45	45	45	45
Sugar	40	40	40	40
Egg	30	30	30	30
Baking powder	1	1	1	1

where APSF is Aleppo pine seed flour, CB is control biscuit (100% wheat flour), B15% is blending ratio 1 (15% Aleppo pine seed flour and 85% wheat flour), B30% is blending ratio 2 (30% Aleppo pine seed flour and 70% wheat flour), and B45% is blending ratio 3 (45% Aleppo pine seed flour and 55% wheat flour).

**Table 2:** Physical and chemical characteristics of of *Pinus halepensis* seeds.

Characteristics	Values
Seed index ^1^	15.43 ± 0.22
Bulk density (g/cm^3^)	0.39 ± 0.01
Moisture ^2^	5.6 ± 0.12
Protein ^2^	28.34 ± 0.10
Lipid ^2^	37.26 ± 0.97
Carbohydrates ^2^	24.81 ± 0.66
Ash ^2^	6.52 ± 0.28
Polyphenols ^3^	663.23 ± 0.05
Flavonoids ^3^	28.10 ± 0.1

Values are mean ± standard deviation of triplicate measurements. ^1^ Seed index = weight of 1000 seeds in grams. ^2^ Values are expressed on a g/100 g. ^3^ Values are expressed on a mg/100 g.

**Table 3:** Minerals of *Pinus halepensis* seeds.

Minerals	Phosphorus	Potassium	Magnesium	Calcium	Sodium	Zinc	Manganese	Iron	Copper
**mg/100 g**	710.9 ± 1.03	270.1 ± 1.02	251.6 ± 0.55	93.3 ± 1.02	61.8 ± 0.96	51.7 ± 1.02	34.5 ± 0.11	73.2 ± 1.10	3.8 ± 0.17

Values are mean ± standard deviation of triplicate measurements.

**Table 4:** Physical properties of biscuits.

	CB	B15%	B30%	B45%
**Diameter (mm)**	44.4 ± 0.44 ^ab^	44.6 ± 0.75 ^b^	43.92 ± 0.37 ^a^	44.48 ± 0.21 ^ab^
**Thickness (mm)**	5.41 ± 0.05 ^b^	5.34 ± 0.02 ^a^	5.32 ± 0.07 ^a^	5.33 ± 0.01 ^a^
**Weight (g)**	8.86 ± 0.54 ^a^	8.97 ± 0.66 ^a^	8.77 ± 0.33 ^a^	9.13 ± 0.37 ^b^
**Spread Ratio**	8.21 ± 0.23 ^a^	8.35 ± 0.27 ^b^	8.28 ± 0.10 ^ab^	8.34 ± 0.41 ^b^
**Color**				
L*	80.77 ± 0.55 ^c^	66.20 ± 1.85 ^b^	52.13 ± 2.94 ^a^	50.33 ± 1.07 ^a^
a*	0.37 ± 0.12 ^a^	2.53 ± 0.42 ^b^	4.63 ± 0.21 ^c^	5.37 ± 0.12 ^d^
b*	29.57 ± 0.42 ^d^	20.37 ± 0.64 ^c^	15.63 ± 0.74 ^a^	17.67 ± 0.40 ^b^

Values are mean ± standard deviation of triplicate measurements. Means in the same line with different superscript are significantly different (*p* ˂ 0.05). Where CB is control biscuit (100% wheat flour), B15% is blending ratio 1 (15% Aleppo pine seed flour and 85% wheat flour), B30% is blending ratio 2 (30% Aleppo pine seed flour and 70% wheat flour), and B45% is blending ratio 3 (45% Aleppo pine seed flour and 55% wheat flour).

**Table 5:** Chemical composition of biscuits.

Characteristics	Biscuits			
	CB	B15%	B30%	B45%
Moisture ^1^	3.41 ± 0.22 ^b^	2.97 ± 0.93 ^b^	3.52 ± 0.74 ^b^	2.27 ± 0.14 ^a^
Carbohydrate ^1^	60.12 ± 0.81 ^c^	51.32 ± 0.22 ^b^	49.11 ± 0.91 ^a^	47.63 ± 0.14 ^a^
Fats ^1^	29.41 ± 0.22 ^a^	31.71 ± 0.15 ^a^	33.22 ± 0.76 ^b^	35.11 ± 0.55 ^b^
Protein ^1^	9.45 ± 0.74 ^a^	11.84 ± 0.26 ^b^	13.79 ± 0.81 ^b^	15.40 ± 0.62 ^c^
Ash ^1^	2.70 ± 0.21 ^a^	3.56 ± 0.63 ^ab^	4.29 ± 0.11 ^b^	4.97 ± 0.17 ^b^
Polyphenols ^2^	3.57 ± 1.10 ^a^	5.58 ± 0.22 ^a^	11.44 ± 1.61 ^b^	31.95 ± 3.18 ^c^
Flavonoids ^2^	0.24 ± 0.04 ^a^	0.54 ± 0.09 ^b^	0.85 ± 0.14 ^c^	1.50 ± 0.21 ^d^
Energy (kcal)	542.97 ± 36 ^a^	538.03 ± 66 ^a^	550.58 ± 70 ^b^	568.11 ± 56 ^b^

Values are mean ± standard deviation of triplicate measurements. Means in the same line with different superscript are significantly different (*p* ˂ 0.05). ^1^ Values are expressed on a g/100 g. ^2^ Values are expressed on a mg/100 g. Where CB is control biscuit (100% wheat flour), B15% is blending ratio 1 (15% Aleppo pine seed flour and 85% wheat flour), B30% is blending ratio 2 (30% Aleppo pine seed flour and 70% wheat flour), and B45% is blending ratio 3 (45% Aleppo pine seed flour and 55% wheat flour).

**Table 6:** Sensory characteristic of biscuits.

Sample	CB	B15%	B30%	B45%
Color	6.55 ± 0.17 ^c^	5.93 ± 0.14 ^c^	5.19 ± 0.44 ^b^	4.19 ± 0.37 ^a^
Taste	7.62 ± 0.51 ^ab^	8.01 ± 0.11 ^b^	8.28 ± 0.24 ^b^	6.54 ± 0.16 ^a^
Crispness	7.12 ± 0.45 ^a^	7.56 ± 1.03 ^ab^	7.93 ± 1.02 ^ab^	8.31 ± 0.10 ^b^
Appearance	8.51 ± 1.06 ^c^	7.51 ± 1.11 ^b^	6.55 ± 0.91 ^b^	5.27 ± 0.23 ^a^
Overall accept	7.91 ± 0.81 ^c^	7.85 ± 0.23 ^c^	6.81 ± 1.37 ^b^	5.33 ± 0.60 ^a^

Values are mean ± standard deviation of 25 measurements. Means in the same line with different superscript are significantly different (*p* ˂ 0.05). Where CB is control biscuit (100% wheat flour), B15% is blending ratio 1 (15% Aleppo pine seed flour and 85% wheat flour), B30% is blending ratio 2 (30% Aleppo pine seed flour and 70% wheat flour), and B45% is blending ratio 3 (45% Aleppo pine seed flour and 55% wheat flour).

## Data Availability

The original contributions presented in the study are included in the article; further inquiries can be directed to the corresponding authors.
